# Comparing dental plaque microbiome diversity of extrinsic black stain in the primary dentition using Illumina MiSeq sequencing technique

**DOI:** 10.1186/s12903-019-0960-9

**Published:** 2019-12-03

**Authors:** Lulu Chen, Qiong Zhang, Yan Wang, Keke Zhang, Jing Zou

**Affiliations:** 1 Department of Pediatric Dentistry, Beijing Stomatological Hospital, Capital Medical University, Beijing, 100050 China; 2 State Key Laboratory of Oral Diseases & National Clinical Research Center for Oral Diseases & Department of Pediatric Dentistry, West China Hospital of Stomatology, Sichuan University, Chengdu, Sichuan China; 3 School and Hospital of Stomatology, Wenzhou Medical University, Wenzhou, Zhejiang, China

**Keywords:** Extrinsic black stain, Dental biofilm, Primary dentition, Illumina MiSeq

## Abstract

**Background:**

Extrinsic black stain (EBS) is characterized by discrete dark dots or lines on the tooth surface. The relationship between EBS and oral microbiota in children remains elusive. The aim of this study was to compare dental plaque microbiome in EBS children with that in EBS-free children in the primary dentition.

**Methods:**

The Illumina MiSeq sequencing technique was utilized in the cross-sectional pilot study to investigate the diversity and composition of the supragingival plaque microbiota from 10 EBS-positive and 10 EBS-free children. The results were analysed with nonparametric Mann-Whitney U test, Pearson Chi-Square test, Fisher’s Exact test and one-way ANOVA tests.

**Results:**

We identified 13 different phyla, 22 classes, 33 orders, 54 families, 105 genera, and 227 species from a total of 52,646 high-quality sequences. Between two groups, no statistical differences were observed in the estimators of community richness and diversity at 97% similarity, as well as in the Unweighted Unifrac principal co-ordinates analysis (PCoA). At the species level, higher level of relative abundance of *Actinomyces naeslundii* and lower level of relative abundance of a species belonging to *Candidate_division_TM7* was observed in dental plaque of EBS-positive subjects, compared to dental plaque of EBS-free subjects (*P* < 0.05). This indicated that some species might be involved in the EBS process.

**Conclusion:**

Changes in dental plaque microbiota is possibly relevant to the process of EBS in the primary dentition.

## Background

**Fig. 1:**
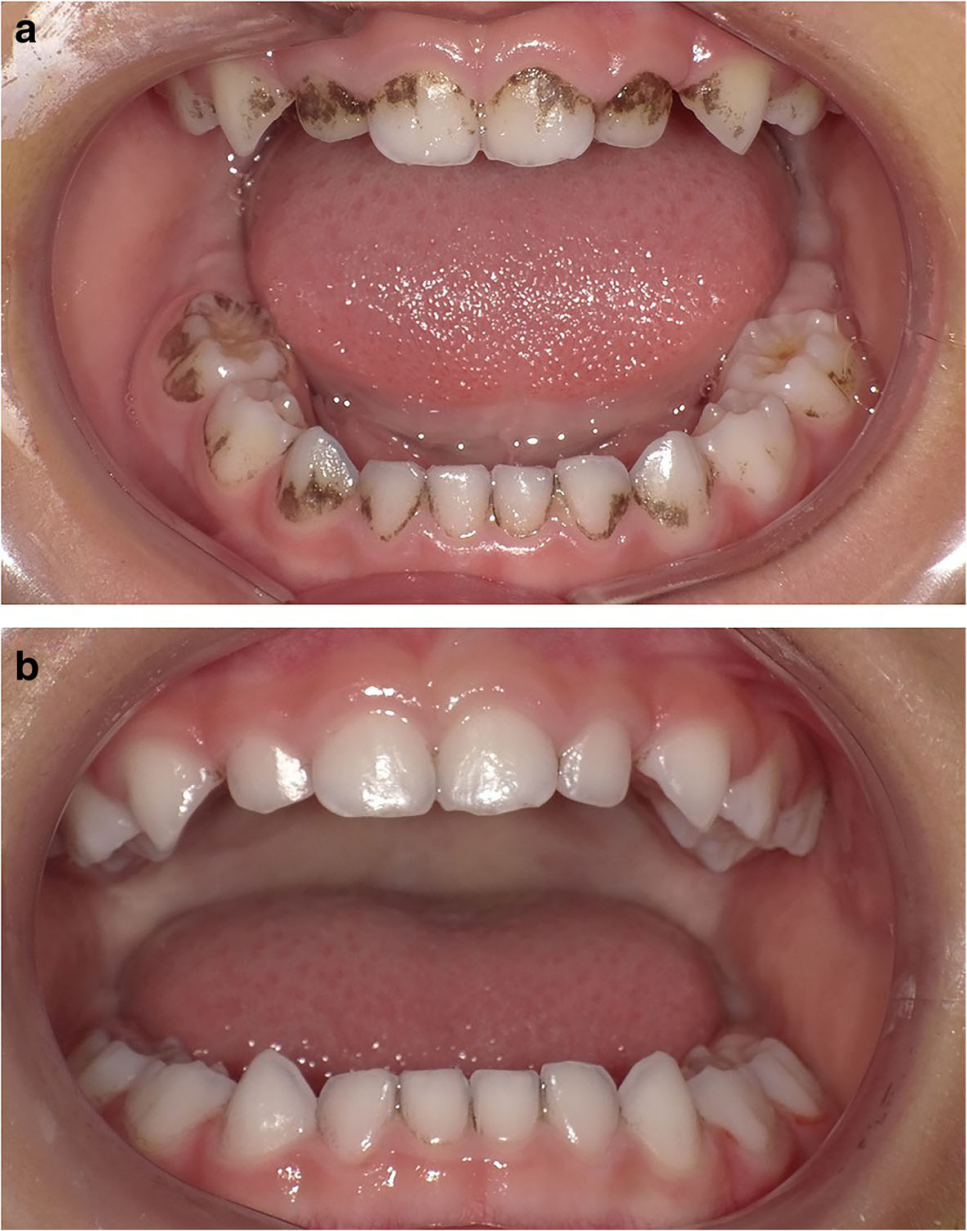
Primary dentition with (**a**) and without (**b**) EBS. **a** The dark pigmentation is in parallel with the third cervical line of the teeth crown. **b** EBS was removed by treatment

Extrinsic black tooth stain (EBS, Fig. 1a), which is defined as dark pigmented extrinsic substance in lines or dots parallel to the third cervical line of the tooth crown in the primary and permanent teeth [1], is often associated with clinical and aesthetic problems [2].

The prevalence of EBS varies between 2.4 and 18% among different populations [3]. Based on the extension of the tooth surface area affected, Gasparetto et al. introduced the following diagnostic scoring system: score 1 corresponded to the presence of pigmented dots or thin lines with incomplete coalescence parallel to gingival margin; score 2 corresponded to continuous pigmented lines that could easily be observed and were limited to half of the cervical third of the tooth surface; score 3 corresponded to the presence of pigmented stains that extended beyond half of the cervical third of the tooth surface [4].

The aetiology of EBS is multi-factorial with chromogens derived from dietary and pigmented elements. Possible causes of EBS include the intake of particular foods and beverages, including tea, coffee, and red wine [5]. In addition, the consumption of fruits, dairy products, and dark colour seasonings, such as soy sauce were reported to promote the development of EBS [6]. Other causes of EBS may include chlorhexidine treatment and the presence of metals such as iron [7].

Previous studies of the microflora of EBS have indicated that black-pigmented bacteria are linked to EBS [8, 9]. It has been reported that Gram-positive rods, especially *Actinomyces israelii* and *Actinomyces naeslundii,* are the predominant cultivable microorganisms of EBS [8]. Saba et al. found that *Actinomyces spp,* had four-fold higher probability of EBS compared to the subjects without these bacteria [9]*.* Interestingly, *Porphyromonas gingivalis* and *Prevotella melaninogenica* were formerly regarded as the main etiologic cause of these pigmentations, however, they were absent in both EBS and control groups. While studying the dental biofilms of patients with or without EBS by multiplex PCR, similar bacterial compositions of EBS-positive and EBS-free groups were identified. However, another research showed similar prevalence of *Actinomyces spp* in the EBS and non-EBS group (19.2% vs. 11.5%, respectively) [10], which did not match the findings reported by Saba et al. The results differ between studies, suggesting that the composition of the EBS-associated microbiota has not been definitively identified.

The oral cavity harbours one of the most complex microbiomes in the body [11]. Traditional culture-dependent studies that focused on mono-species or certain types of microbiome underestimated the mixed species oral microbial communities [12], therefore, efforts to investigate microbial diversity increasingly relied on cultivation-independent, molecular approaches [13]. DAN-based methods, such as next-generation FLX+ and, Illumina pyrosequencing methods, combined with curated gene databases, such as the Human Oral Microbiome Database [14], are comprehensive methods to analyse the microbiota. MiSeq, Illumina’s next generation sequencing techonology, has been wildly used nowadays as it is intuitively multi-function, including amplification, sequencing and data analysis. By using MiSeq, it is capable of identifying the bacteria composition from complex environments in a culture-free way [15].

The aim of the present study was to evaluate the plaque microbiota in children with high prevalence of EBS and who had no EBS by using the Illumina MiSeq, thereby identifying a potential role for the microbiome in EBS among children. While we expand our efforts to identify the dental plaque microbiome diversity of EBS in children with primary dentition will greatly aid in our understanding of the potential role of bacteria in the process of EBS as limited research has been conducted regarding microbiology by next-generation sequencing methods.

## Materials and methods

### Experimental subjects

A total of 100 children in primary dentition were recruited for this cross-sectional pilot study by the Department of Paediatric Dentistry, West China Hospital of Stomatology, Sichuan University (Chengdu, China). The oral health status of all participants was determined by a dentist who performed a full-mouth clinical examination that included inspection of the teeth, oral mucosa, and periodontal tissues.

Based on the clinical examination according to criteria defined by Gasparetto et al., 10 children who presented EBS (sore 2–3) pigmented stains that were limited to or extended beyond half of the cervical third of the tooth surface and were negative to other extrinsic discoloration (e.g. brown, green or orange stain), were included in this study (Group BP).

For each child with EBS, a gender-, age-, and dentition status-matched EBS-free counterpart (10 children in total, Group BFP) was recruited as a control. All children didn’t have antibiotics or any type of polishing within 3 months prior to the start of the study. Children with other oral disease, such caries, periodontitis or intrinsic BS, or systematic diseases were excluded from the study. Inclusion and exclusion criteria are listed in Table 1.

**Table 1:** Enrollment criteria

Inclusion criteria	Exclusion criteria
EBS-positive subjects	
Having at least 10 teeth with EBS in the oral cavity, with score 2–3	Receiving antibiotics within 3 months before the study
3–5 years of age (primary dentition)	Receiving any kind of polishing within 3 months before the study
Free of systemic diseases	Having intrinsic black stain
Free of caries, gingivitis or periodontitis, etc.	
Written informed consent	
EBS-free subjects	
Age, gender and dentition status comparable with EBS-positive patients	Receiving antibiotics within 3 months before the study
Free of any kind of teeth extrinsic stain	Receiving any kind of polishing within 3 months before the study
Free of systemic diseases	Having intrinsic black stain
Free of caries, gingivitis or periodontitis, etc.	
Written informed consent	

### Sample collection

Plaque samples were collected from supragingival tooth surfaces using sterilized curettes. For Group BP, samples were collected from the labial or buccal surfaces of EBS teeth and pooled. For Group BFP, specimens were obtained from the corresponding positions and surfaces of the teeth. Pooled plaque samples were removed from the curette by agitation in 700 ml of Tries-EDTA (TE) buffer (10 mM Tris-Cl [pH 7.5] and 1 mM EDTA) and stored at − 80 °C for further studies.

### DNA extraction and PCR amplification

The total bacterial genomic DNA was extracted from pooled plaque samples using the QIAamp DNA Micro Kit (Qiagen, Hilden, Germany) according to manufacturer’s protocols. The V3-V4 hypervariable region of the bacteria 16S ribosomal RNA gene were amplified by PCR using primers 338F 5′-barcode-ACTCCTACGGGAGGCAGCAG-3′ and 806R 5′-GGACTACHVGGGTWTCTAAT − 3′, in which the barcode represented an eight-base sequence that was unique to each sample [16]. PCR reactions were performed in triplicate in a total of 20 μl mixture, containing 4 μl of 5 × FastPfu Buffer, 2 μl of 2.5 mM dNTPs, 0.8 μl of each primer (5 μM), 0.4 μl of FastPfu Polymerase, and 10 ng of template DNA. The PCR conditions were as follows: 94 °C for 3 min for DNA denaturation, followed by 27 cycles (94 °C for 30 s, 50 °C for 30 s, and 72 °C for 45 s), and a final extension for 10 min at 72 °C. The amplicons of three replicates were pooled to obtain sufficient PCR products.

### Illumina MiSeq sequencing

After PCR, amplicons were separated using 2% agarose gels and extracted and purified using the AxyPrep DNA Gel Extraction Kit (Axygen Biosciences, Union City, CA, USA). Then, amplicons were quantified using QuantiFluor™ -ST (Promega, Madison, WI, USA) according to the manufacturer’s instructions. Purified amplicons were pooled in equimolar and paired-end sequenced (2 × 250) on an Illumina MiSeq platform (Majorbio, Shanghai, China) according to the standard protocols. The raw data were deposited into the NCBI Sequence Read Archive (SRA) database for evaluation.

### Processing of sequencing data

Raw FASTQ files were de-multiplexed, quality-filtered using QIIME (version 1.9.1) using the following criteria: (i) 300 bp reads were truncated at any site receiving an average quality score < 20 over a 50 bp sliding window. Truncated reads shorter than 50 bp were eliminated; (ii) exact barcode matching reads (2 nucleotide mismatch in primer matching) containing ambiguous characters were removed; (iii) based to their overlap sequence, only sequences with an overlap of > 10 bp were assembled. Reads that could not be assembled were eliminated.

UPARSE software (version 7.1 http://drive5.com/uparse/) was used to cluster quality-checked sequences into Operational Taxonomic Units (OTUs) at a similarity threshold of 97% for production of OTUs and removed using UCHIME [17].

The taxonomy of each 16S rRNA gene sequence was analysed by Ribosomal Database Project (RDP) Classifier (http://rdp.cme.msu.edu/) against the SILVA (SSU123) 16S rRNA database using a confidence threshold of 70% [18]. The relative abundance of bacterial genera was calculated using QIIME software [19], and rarefaction curve analysis, community richness (ACE, Chao1), and community diversity indices (Simpson, Shannon diversity index) were determined by the MOTHUR program at a 97% similarity level. To examine dissimilarities in community composition, principal co-ordinates analysis (PCoA) in QIIME was performed, which was used to compare groups of samples based on weighted and weighted UniFrac distance metrics [20]. Using Vegan packages in R, heat map profiles were generated.

### Statistical analysis

The independent sample nonparametric Mann-Whitney U test, Pearson Chi-Square test, Fisher’s Exact test and one-way ANOVA test were performed using SPSS version 23.0 (SPSS Inc., Chicago, IL, USA).

## Results

### Sequencing results and diversity indices

In this study, a total of 552,646 high-quality sequences were produced, with an average of 27,632 sequences per sample. Summary information is shown in Table 2, and detailed characteristics of each sample are presented in Additional file 1: Table S1. The numbers of OTUs (3% dissimilarity) and detailed characteristics for each sample are shown in Additional file 1: Table S1. The average OTUs of Group BP and BFP were 153.7 ± 32.2 and 156.4 ± 24.6, respectively (*P* > 0.05). The rarefaction curves of Group BP and BFP reached a saturation plateau, indicating that the data volume of sequenced reads was reasonable (Additional file 2: Figure S1). Comparisons of the estimators of community richness (Chao1 and Ace) and diversity (Shannon index and Simpson indices) at 97% similarity between two groups are shown in Table 3. One-way ANOVA analysis did not show significant differences in α-diversity between Group BP and BFP (*P* > 0.05).

**Table 2:** Demographic and oral health information of EBS-positive and EBS-free subjects

Variables	Characteristics	BP (*n* = 10)	BFP (*n* = 10)	*P* value
Age (years)	Mean ± SD	4.34 ± 0.81	4.45 ± 0.83	0.767^a^
Gender	Male	5	5	1.000^b^
	Female	5	5	
Extrinsic black stain condition	EBS-positive		10	1.000^c^
	EBS-free	10		

*BP* Plaque samples from EBS-positive subjects. *BFP* Plaque samples from EBS-free subjects

^a^ Independent sample non-parametric Mann-Whitney U test was used

^b^ Pearson Chi-Square test was used

^c^ Fisher’s Exact test was used

**Table 3:** Comparison of richness and diversity estimates of 16S rRNA gene libraries at 97% similarity between Group BP and BFP.

Variables	BP (*n* = 10)	BFP (*n* = 10)	P^*^ value
OTUs	153.7 ± 32.3	156.4 ± 24.6	0.831
Chao1^a^	176.9 ± 32.2	173.2 ± 26.2	0.784
ACE^b^	174.5 ± 31.2	174.1 ± 24.0	0.974
Simpson^c^	0.075 ± 0.048	0.067 ± 0.028	0.597
Shannon^d^	3.38 ± 0.56	3.46 ± 0.31	0.672

Values are presented as the Mean ± SD. *BP* Plaque samples from EBS-positive subjects. *BFP* Plaque samples from EBS-free subjects

^a,b^Richness estimators (Chao 1 and ACE) were calculated using MOTHUR

^c^A higher number indicates less diversity

^*^One-way ANOVA test was used

### Taxonomy-based comparisons of oral microbiota between group BP and BFP

To investigate whether EBS was correlated with potential changes in the abundance of specific bacterial taxa, the relative abundance of taxa was compared.

**Fig. 2:**
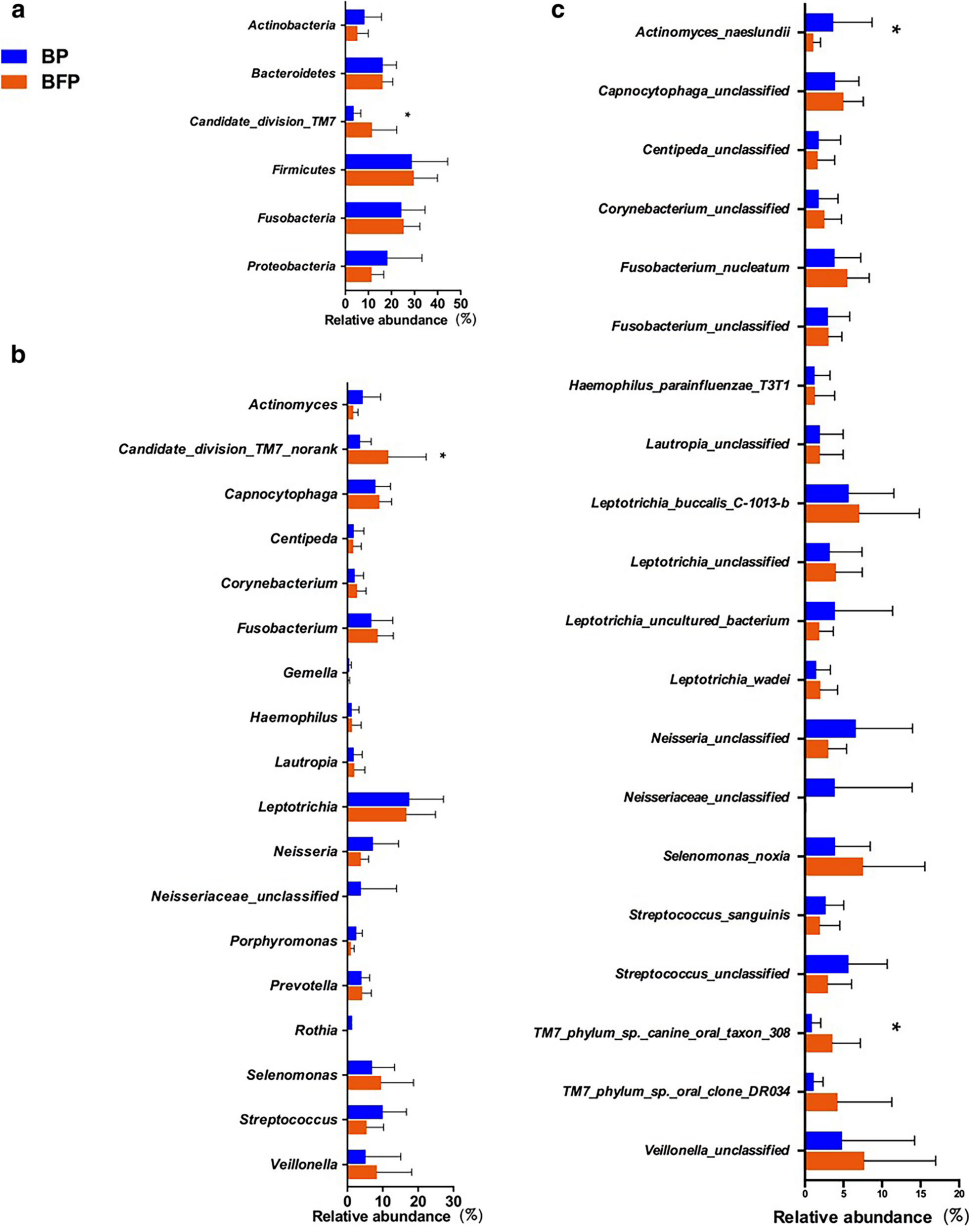
Taxonomic distribution of supragingival plaque samples. BP: Plaque samples from EBS-positive subjects. BFP: Plaque samples from EBS-free subjects. **a** Phylum distribution of all samples. **b** Genus distribution of all samples. **c** Species distribution of all samples

Sequences from these samples were classified into 13 different phyla, 22 classes, 33 orders, 54 families, 105 genera, and 227 species. The overall microbiota structure for each group at the phylum level is presented in Fig. 2a. Out of a total of 13 phonotypes, six different phyla (*Formicates, Fusobacteria, Proteobacteria, Bacteroidetes, Actinobacteria,* and *Candidate_division_TM7*) were common, and comprised a considerable proportion of 99.5%. *Firmicutes* were strongly enriched, accounting for 28.9% of the total sequences in Group BP, followed by *Fusobacteria* (24.4%), *Proteobacteria* (18.3%), *Bacteroidetes* (16.2%), *Actinobacteria* (8.3%), and *Candidate_division_TM7* (3.6%). Moreover, microbiota in Group BFP primarily included *Firmicutes* (29.7%), *Fusobacteria* (25.3%), *Bacteroidetes* (16.1%), *Candidate_division_TM7* (11.6%), *Proteobacteria* (11.4%), and *Actinobacteria* (5.3%).

Statistical differences between the two groups were found only for *Candidate_division_TM7* (*P* < 0.05). The relative abundance of the dominant genera identified in the two groups is presented in Fig. 2b (only those with a relative abundance of > 1% are listed). In Group BP, *Actinomyces, Candidate_division_TM7_norank, Capnocytophaga, Centipeda, Corynebacterium, Porphyromonas,* and *Fusobacterium* exhibited a relatively higher abundance compared to Group BFP. *Gemella, Haemophilus, Lautropia, Leptotrichia, Neisseria,* and *Neisseriaceae_unclassified* were relatively more abundant in Group BFP. Statistically significant differences between Group BP and BFP were only found in *genus Candidate_division_TM7_norank* (*P* < 0.05).

Figure 2c showed the relative abundance of the major bacterial community at the species levels (only those above 1% in relative abundance are listed). Our data indicated that the bacterial community composition showed a few differences between Group BP and BFP. For example, species *Actinomyces naeslundii* were significantly more abundant in Group BP compared to Group BFP, whereas species *TM7_phylum_sp._canine_oral_taxon_308* was significantly less abundant in Group BP compared to Group BFP (*P* < 0.05).

The top 100 abundant genera among the 20 samples were displayed based on the heat map of the genus-level plaque composition (Fig. 4), and indicated that between Group BP and BFP most genera were shared. In both groups, the dominant genera were *Neisseria, Streptococcus, Fusobacterium, Capnocytophaga,* and *Veilonella*. Moreover, minor genera, such as *Gemella, Lactobacillales,* and *Stomatobaculum* were found in both groups. However, some genera, including *genoceanbacillus*, *myroidesu*, *brochotrix*, were predominantly found in Group BFP.

### Comparison of beta diversity between groups

**Fig. 3:**
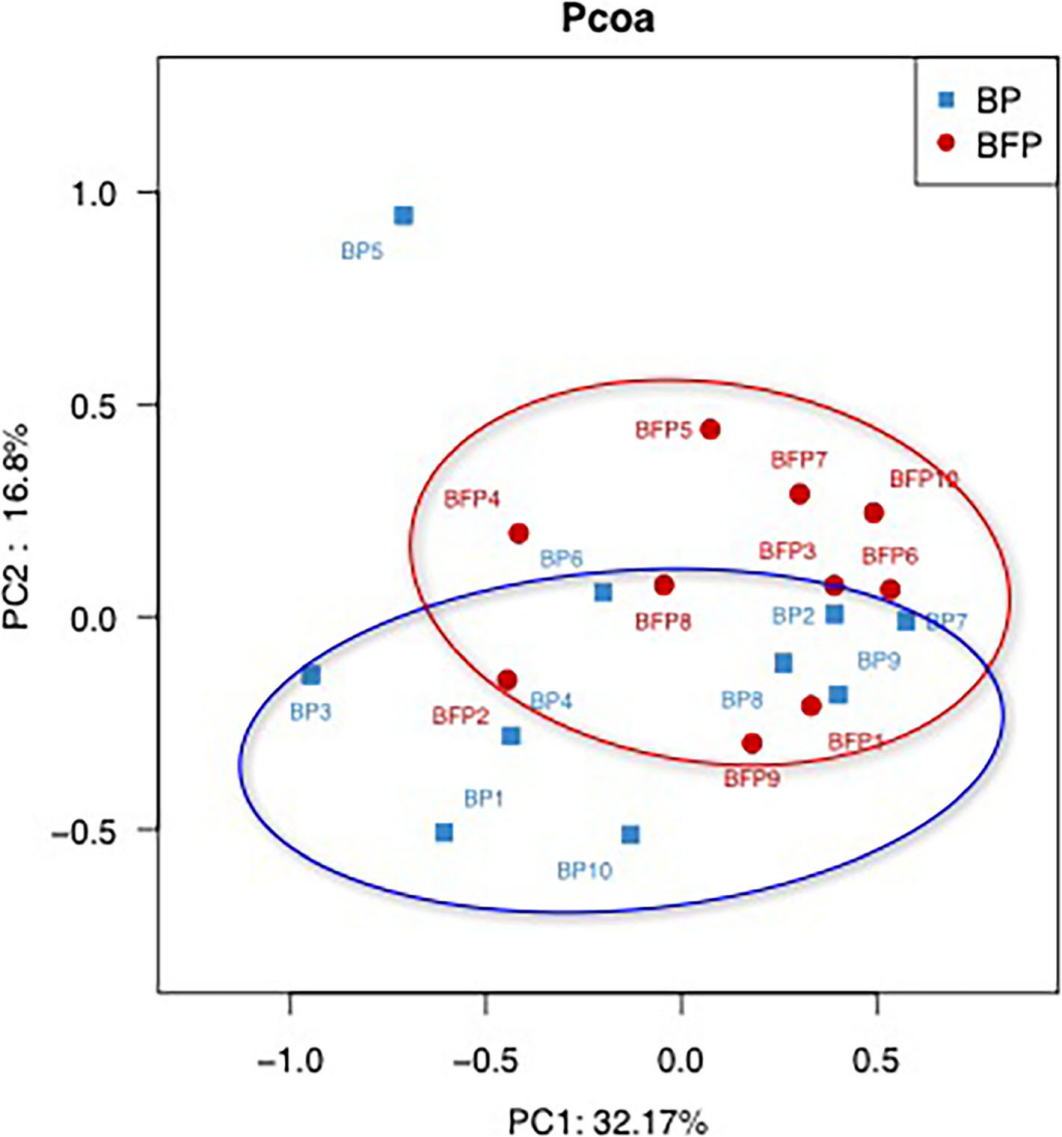
Unweighted Unifrac PCoA analysis. For each sample, the first two principal coordinates (PCo1 and PCo2) from the principal coordinate analysis of weighted UniFrac are plotted. The variance as calculated by PCoA is indicated in parentheses on the axes. BP: Plaque samples from EBS-positive subjects. BFP: Plaque samples from EBS-free subjects

In this study, PCoA based on weighted UniFrac matrix was used to identify community structure differences (Fig. 3), and showed that the oral microbial structure of Group BP differed from that of Group BFP. On the primary axes, the PCoA ordination did not reveal strong grouping of disease and health, however, a segregation trend for EBS-positive patients and EBS-free subjects was observed.

**Fig. 4:**
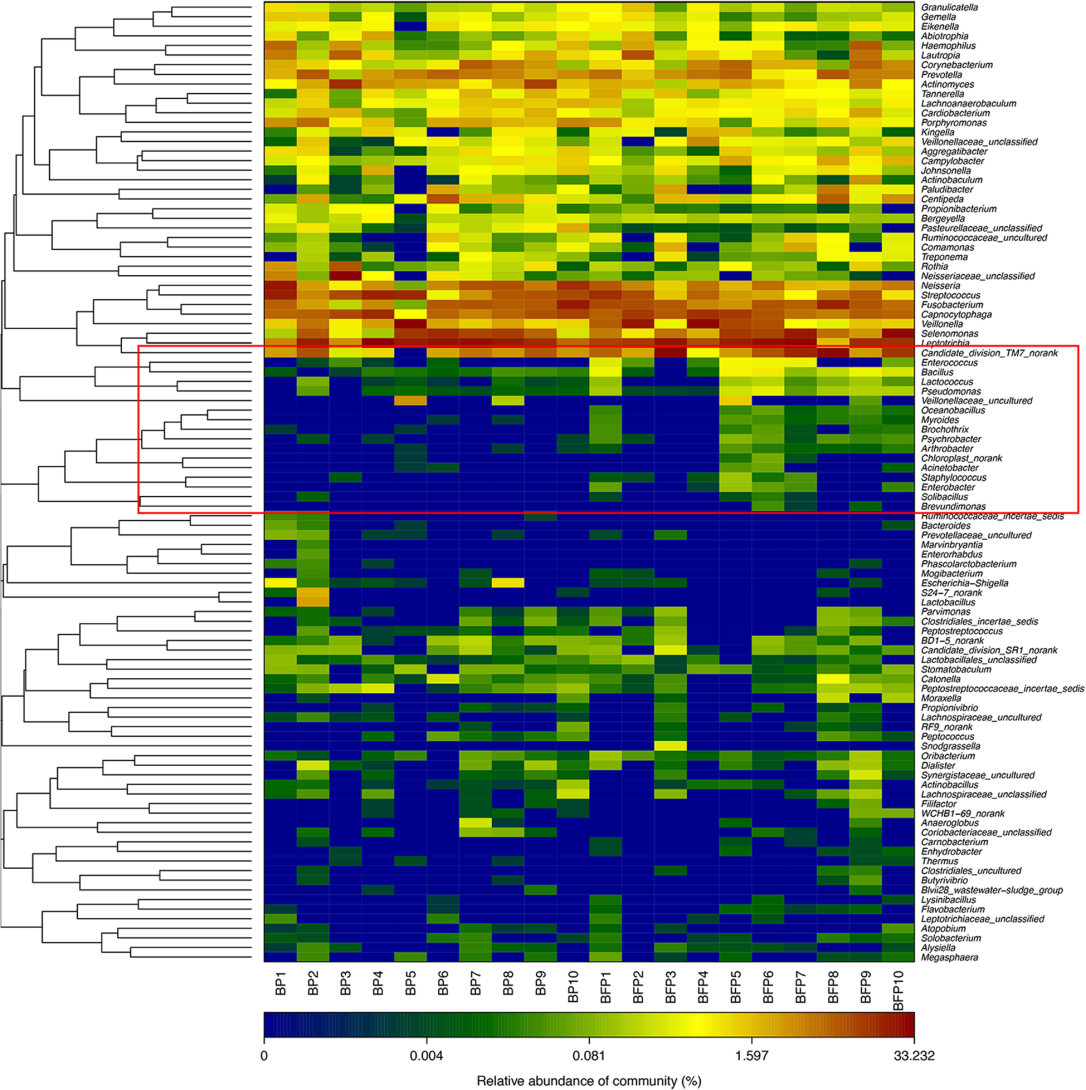
Bacterial distribution of the top 100 most abundant genera among the 20 samples. Heat map analysis was performed based on the hierarchical clustering solution (Bray-Curtis) distance metric. Columns represent the sample ID, and rows represent the predominant bacterial genera. Variables of clustering are presented on the vertical axis. The relative values for bacterial genera are indicated by colour intensity, the legend is indicated at the top right corner. BP: Plaque samples from EBS-positive subjects. BFP: Plaque samples from EBS-free subjects

## Discussion

The human mouth harbours a highly diverse bacterial community, however, only about 50% of oral microorganisms can be cultivated [21]. In previous studies, the microbiology of EBS has been investigated. However, these studies were impeded by the limitation of conventional culture-dependent approaches [12], or only focused on a few selected bacteria [10, 22], without characterizing the overall community of oral microbiota in EBS patients. Accordingly, we used high-throughput DNA sequencing technique, Illumina Miseq to investigate the holistic pattern of dental biofilm microbiome. Moreover, bacteria with low abundance or not-yet-cultivated members could be investigated in children with or without EBS [23].

No statistical differences were found from the Chao1 and Ace estimator between Group BP and BFP. In addition, a high similarity of Shannon and Simpson index was observed between the two groups, demonstrating that similar dental biofilm microbial community richness and diversity in these two groups. These findings were in line with the recent findings reported by Yue Li, et al. [24]. The difference was not statistically significant, which was likely due to the small sample size of our pilot study, however a segregation trend was observed in the Unweighted Unifrac PCoA analysis. Additional research, with larger study groups, is required to verify the role of EBS in shaping the overall pattern of the dental biofilm microbiome community.

In our study, we haven’t observed significant differences in alpha and beta diversity, however, we did identify potential microbiota associated with EBS in children. Our findings indicated that *Candidate_division_TM7* was less abundant in the supragingival plaque of Group BP compared to Group BFP from the phylum down to the species level. To our knowledge, this is the first study that reported significant differences between the prevalence of *Candidate_division_TM7* in children with or without EBS.

*Candidate_division_TM7*, initially discovered from the German peat bog by environmental sequencing data, represents one of uncultivable bacterial divisions [25]. Using next-generation sequencing strategies, highly divergent members of this division were identified in a range of cosmopolitan habitats, including terrestrial, aquatic, and human body sites [26]. Recently, the ability to modulate the oral biofilm formation of TM7 was reported. The morphology of TM7 in dual-species biofilms with different types of bacteria varied, as did the morphological characteristics of biofilm. It was speculated that the interactions between TM7 and other microorganisms affected the oral microbial community and the process of infectious oral diseases [27]. In present study, *Candidate_division_TM7* was reduced in Group BP compared to Group BFP, however the mechanism remains elusive. We hypothesized a possible correlation between the decrease in *Candidate_division_TM7* and the development of EBS through complicated bacterial interactions. Further studies are required to clarify the underlying mechanism.

Furthermore, our study is consistent with previous reports [22, 24] that *Actinomyces naeslundii* was more prevalent in supragingival plaque of EBS patients. *A. naeslundii* is one of the resident microbiota in the human mouth and plays a considerable role in dental biofilm formation [28]. Sarkonen et al. [29] demonstrated that other Actinomyces species, including *A. odontolyticus*, could form brown to black pigmentation. In addition, Reid et al. considered that the EBS was formed as a result of the reaction between ferric ions in saliva and the hydrogen sulfide produced by microorganisms (for example *Actinomycetes*) [30].

*Prevotella spp*. was identified as the predominant bacteria of black pigmentations in earlier studies [31], while, our study showed no significant differences regarding of the prevalence of *Prevotella spp*. between Group BP and BFP, which is consistent with most recent studies [9, 10, 22].

## Conclusion

Based on the results of the current study, our data demonstrated that children in primary dentition with or without EBS share a similar dental biofilm microbial community. *Candidate_division_TM7* and *Actinomyces naeslundii* may possibly be involved in the presence of EBS. Although the sample size in our study was relatively small and further experiments need to be done, our findings represented a comprehensive picture of the microbial structure of children with EBS.

## Supplementary information

**Additional file 1: Table S1.** The number of OTUs, species richness, and diversity estimates in each supragingival plaque microbiome.

**Additional file 2: Figure S1.** Rarefaction curves of unique OTUs at a 97% threshold (a box graph at the rarefied sequence number).

## Data Availability

The datasets used and/or analysed during the current study are available from the corresponding author on reasonable request.

## Abbreviations

EBS: Extrinsic black stain
BP: Plaque samples from EBS-positive subjects
BFP: Plaque samples from EBS-free subjects
OTUs: Operational Taxonomic Units
PCoA: Principal co-ordinates analysis
TE: Tries-EDTA
