# Supplementary material for: Comparing dental plaque microbiome diversity of extrinsic black stain in the primary dentition using Illumina MiSeq sequencing technique
Source: BMC Oral Health. 2019 Dec 3;19:269. doi: 10.1186/s12903-019-0960-9 (PMC6892020; doi:10.1186/s12903-019-0960-9)
Supplement: Supplementary file 1 — Additional file 1: Table S1. The number of OTUs, species richness, and diversity estimates in each supragingival plaque microbiome. [file 12903_2019_960_MOESM1_ESM.docx]

**Table S1** The number of OTUs, species richness, and diversity estimates in each supragingival plaque microbiome.

|  | Reads | OTU | ACE | Chao | Coverage | Shannon | Simpson |
| --- | --- | --- | --- | --- | --- | --- | --- |
| BP1 | 39280 | 139 | 151 | 158 | 0.999516 | 3.22 | 0.0776 |
| BP2 | 19949 | 208 | 224 | 235 | 0.998747 | 4.16 | 0.0246 |
| BP3 | 29901 | 113 | 164 | 152 | 0.999097 | 2.59 | 0.1483 |
| BP4 | 32594 | 154 | 186 | 187 | 0.998988 | 3.14 | 0.0989 |
| BP5 | 41944 | 112 | 127 | 126 | 0.999595 | 2.41 | 0.1625 |
| BP6 | 44458 | 120 | 133 | 135 | 0.999663 | 3.34 | 0.0691 |
| BP7 | 19053 | 166 | 179 | 193 | 0.99895 | 3.78 | 0.0435 |
| BP8 | 40062 | 182 | 203 | 205 | 0.999376 | 3.81 | 0.0381 |
| BP9 | 22844 | 165 | 177 | 175 | 0.999168 | 3.78 | 0.0377 |
| BP10 | 29776 | 178 | 201 | 203 | 0.99906 | 3.61 | 0.0511 |
| BFP1 | 25247 | 171 | 179 | 178 | 0.999445 | 3.79 | 0.0381 |
| BFP2 | 21043 | 120 | 140 | 143 | 0.999002 | 3.09 | 0.0844 |
| BFP3 | 28156 | 171 | 187 | 190 | 0.999219 | 3.58 | 0.0546 |
| BFP4 | 32738 | 121 | 154 | 137 | 0.999359 | 3.2 | 0.09 |
| BFP5 | 17127 | 149 | 161 | 162 | 0.998949 | 3.57 | 0.0505 |
| BFP6 | 17865 | 155 | 170 | 174 | 0.99888 | 3.7 | 0.0415 |
| BFP7 | 33028 | 143 | 158 | 156 | 0.999455 | 2.93 | 0.1212 |
| BFP8 | 18256 | 180 | 206 | 206 | 0.998357 | 3.4 | 0.0817 |
| BFP9 | 20804 | 197 | 218 | 219 | 0.99875 | 3.9 | 0.0339 |
| BFP10 | 18521 | 157 | 168 | 167 | 0.999082 | 3.44 | 0.0741 |

**Figure S1** Rarefaction curves of unique OTUs at a 97% threshold (a box graph at the rarefied sequence number).
